# Supplementary material for: Dual Transcriptome Analysis Reveals That ChATG8 Is Required for Fungal Development, Melanization and Pathogenicity during the Interaction between Colletotrichum higginsianum and Arabidopsis thaliana
Source: Int J Mol Sci. 2023 Feb 22;24(5):4376. doi: 10.3390/ijms24054376 (PMC10002072; doi:10.3390/ijms24054376)
Supplement: Supplementary file 1 [file ijms-24-04376-s001.zip › Supplementary file/Supplementary Materials.pdf]

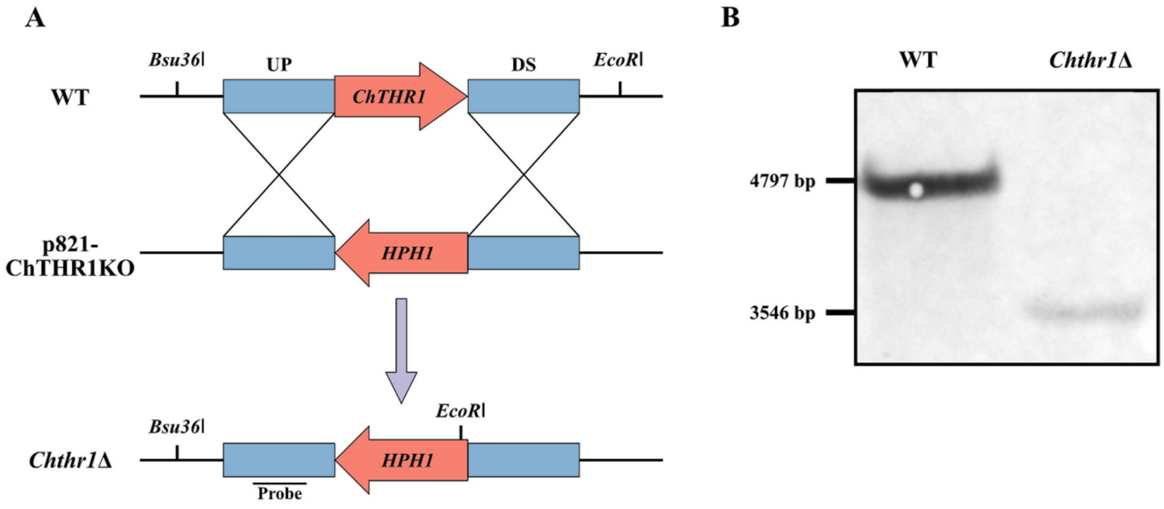

Figure S1. Targeted gene deletion of *ChTHR1* in *C. higginsianum*. (A) Strategic map of gene disruption vector construction and sites for restriction enzymes at the *ChTHR1* genomic region. A bold line below the disruption construct represents the sequence used as a probe in Southern blot analysis. (B) Southern blot analysis of the WT and *Chthr1*Δ mutants. Genomic DNA was digested with *Bsu36*I and *EcoR*I, and subsequently separated in 0.8% agarose gel. Blot was hybridized with the probe amplified from genomic DNA of the WT.

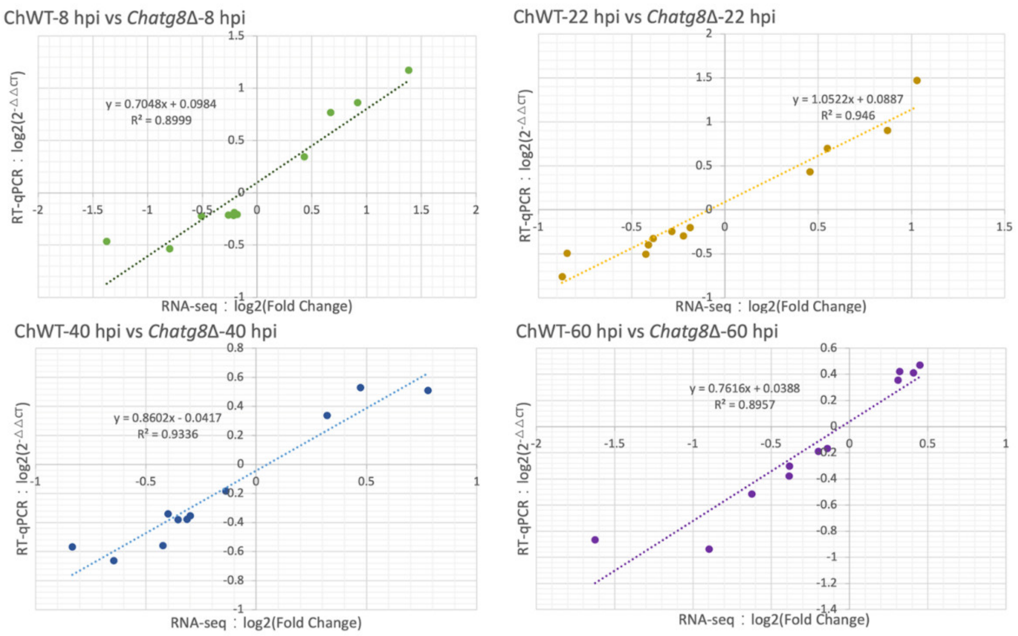

Figure S2. Correlations of the fold changes of six DEGs each from *C. higginsianum* and *A. thaliana* determined by RNA-seq and RT-qPCR analysis.

Table S1. Summary of RNA-Seq data and sequence assembly. *C. higginsianum* (Reference Genes: 14651; Sequenced Reference Genes: 13677, 93.35%; Sequenced Novel Genes: 1378; Sequenced Total_Genes: 15055, 93.92%) and *A. thaliana* (Reference Genes: 27416; Sequenced Reference Genes: 23670, 86.34%; Sequenced Novel Genes: 286; Sequenced Total_Genes: 23956, 86.48%).

Sample	Raw Reads	Clean Reads (%)	Raw Data (bp)	Clean Data (bp)	Q20 (%)	Q30 (%)	*C. higginsianum*			*A. thaliana*		
							Sequenced Reference Genes	Sequenced Novel Genes	Sequenced Total Genes	Sequenced Reference Genes	Sequenced Novel Genes	Sequenced Total Genes
ChWT-8 hpi-1	95715576	95607716 (99.89%)	14357336400	14121532219	13769677256 (97.51%)	13104671951 (92.80%)	10211 (69.69%)	619 (44.92%)	10830 (67.57%)	20717 (75.57%)	275 (96.15%)	20992 (75.78%)
ChWT-8 hpi-2	90214224	90112506 (99.89%)	13532133600	13432281220	13095002760 (97.49%)	12455288188 (92.73%)	9179 (62.65%)	432 (31.35%)	9611 (59.96%)	20518 (74.84%)	270 (94.41%)	20788 (75.04%)
ChWT-8 hpi-3	63256348	63181086 (99.88%)	9488452200	9421649854	9142452445 (97.04%)	8651933548 (91.83%)	9124 (62.28%)	406 (29.46%)	9530 (59.45%)	20433 (74.53%)	270 (94.41%)	20703 (74.73%)
*Chatg8*Δ-8 hpi-1	79490366	79389750 (99.87%)	11923554900	11848474748	11546266278 (97.45%)	10979434460 (92.67%)	9305 (63.51%)	432 (31.35%)	9737 (60.75%)	20507 (74.80%)	271 (94.76%)	20778 (75.01%)
*Chatg8*Δ-8 hpi-2	79566370	79472186 (99.88%)	11934955500	11855442126	11552373824 (97.44%)	10983778041 (92.65%)	9273 (63.29%)	432 (31.35%)	9705 (60.55%)	20462 (74.64%)	269 (94.06%)	20731 (74.84%)
*Chatg8*Δ-8 hpi-3	82019434	81924600 (99.88%)	12302915100	12187079104	11889821109 (97.56%)	11326041763 (92.93%)	9647 (65.85%)	498 (36.14%)	10145 (63.29%)	20653 (75.33%)	269 (94.06%)	20922 (75.53%)
ChWT-22 hpi-1	97034188	96938994 (99.90%)	14555128200	14417167196	14029929722 (97.31%)	13313926982 (92.35%)	9345 (63.78%)	454 (32.95%)	9799 (61.13%)	20952 (76.42%)	275 (96.15%)	21227 (76.63%)
ChWT-22 hpi-2	88555308	88464200 (99.90%)	13283296200	13169889069	12822481654 (97.36%)	12177168183 (92.46%)	8453 (57.70%)	346 (25.11%)	8799 (54.89%)	20672 (75.40%)	270 (94.41%)	20942 (75.60%)
ChWT-22 hpi-3	72358928	72305502 (99.93%)	10853839200	10710036206	10471893924 (97.78%)	10004697432 (93.41%)	9673 (66.02%)	494 (35.85%)	10167 (63.43%)	20684 (75.44%)	277 (96.85%)	20961 (75.67%)
*Chatg8*Δ-22 hpi-1	77768214	77680294 (99.89%)	11665232100	11537014741	11234077127 (97.37%)	10670559433 (92.49%)	9732 (66.43%)	535 (38.82%)	10267 (64.05%)	20738 (75.64%)	271 (94.76%)	21009 (75.84%)
*Chatg8*Δ-22 hpi-2	81762346	81674452 (99.89%)	12264351900	12166769470	11854587811 (97.43%)	11268327124 (92.62%)	9516 (64.95%)	539 (39.11%)	10055 (62.73%)	20568 (75.02%)	273 (95.45%)	20841 (75.23%)
*Chatg8*Δ-22 hpi-3	78869142	78784002 (99.89%)	11830371300	11745636651	11443187620 (97.43%)	10876602946 (92.60%)	9439 (64.43%)	492 (35.70%)	9931 (61.96%)	20516 (74.83%)	270 (94.41%)	20786 (75.03%)

ChWT-40 hpi-1	75795496	75713222 (99.89%)	11369324400	11285769073	10981729143 (97.31%)	10424212710 (92.37%)	9692 (66.15%)	525 (38.10%)	10217 (63.74%)	20938 (76.37%)	271 (94.76%)	21209 (76.56%)
ChWT-40 hpi-2	73596550	73527540 (99.91%)	11039482500	10968636339	10696685533 (97.52%)	10186300040 (92.87%)	9818 (67.01%)	535 (38.82%)	10353 (64.59%)	21017 (76.66%)	273 (95.45%)	21290 (76.85%)
ChWT-40 hpi-3	59280912	59221372 (99.90%)	8892136800	8790719466	8567481013 (97.46%)	8151187861 (92.72%)	9160 (62.52%)	461 (33.45%)	9621 (60.02%)	20565 (75.01%)	269 (94.06%)	20834 (75.21%)
*Chatg8*Δ-40 hpi-1	76507870	76427090 (99.89%)	11476180500	11405522247	11110025056 (97.41%)	10558964946 (92.58%)	9106 (62.15%)	440 (31.93%)	9546 (59.55%)	20429 (74.51%)	274 (95.80%)	20703 (74.73%)
*Chatg8*Δ-40 hpi-2	77155780	77072802 (99.89%)	11573367000	11491538600	11195665317 (97.43%)	10640739123 (92.60%)	8788 (59.98%)	447 (32.44%)	9235 (57.61%)	20567 (75.02%)	267 (93.36%)	20834 (75.21%)
*Chatg8*Δ-40 hpi-3	83400778	83315682 (99.90%)	12510116700	12421517638	12128893059 (97.64%)	11568620128 (93.13%)	8937 (61.00%)	461 (33.45%)	9398 (58.63%)	20655 (75.34%)	273 (95.45%)	20928 (75.55%)
ChWT-60 hpi-1	78276806	78175806 (99.87%)	11741520900	11615432099	11313230463 (97.40%)	10760244358 (92.64%)	12051 (82.25%)	1020 (74.02%)	13071 (81.55%)	20996 (76.58%)	266 (93.01%)	21262 (76.75%)
ChWT-60 hpi-2	80758858	80675568 (99.90%)	12113828700	12018443675	11720063668 (97.52%)	11159313402 (92.85%)	11089 (75.69%)	766 (55.59%)	11855 (73.96%)	20993 (76.57%)	272 (95.10%)	21265 (76.76%)
ChWT-60 hpi-3	95345372	95232228 (99.88%)	14301805800	14173946131	13781325348 (97.23%)	13069065237 (92.20%)	11352 (77.48%)	847 (61.47%)	12199 (76.11%)	20947 (76.40%)	271 (94.76%)	21218 (76.59%)
*Chatg8*Δ-60 hpi-1	71040494	70952912 (99.88%)	10656074100	10563745393	10265260915 (97.17%)	9723366735 (92.04%)	8164 (55.72%)	388 (28.16%)	8552 (53.35%)	20528 (74.88%)	268 (93.71%)	20796 (75.07%)
*Chatg8*Δ-60 hpi-2	73369986	73282734 (99.88%)	11005497900	10922707207	10654317783 (97.54%)	10148614576 (92.91%)	7558 (51.59%)	337 (24.46%)	7895 (49.25%)	20439 (74.55%)	267 (93.36%)	20706 (74.75%)
*Chatg8*Δ-60 hpi-3	71288290	71206044 (99.88%)	10693243500	10615886799	10349987494 (97.50%)	9846294440 (92.75%)	7419 (50.64%)	321 (23.29%)	7740 (48.29%)	20439 (74.55%)	270 (94.41%)	20709 (74.76%)

Note: **Q20/30 (%): Percentage of bases whose clean data mass value is greater than or equal to 20/30**

Table S2. 21 candidate genes of plant hormone signal transduction in Figure 6.

Plant hormone		Gene ID	Description
**Auxin**	**ARF**	AT1G19850	Auxin-responsive factor AUX/IAA-related
	**GH3**	AT4G37390	Auxin-responsive GH3 family protein
		AT1G28130	Auxin-responsive GH3 family protein
		AT5G54510	Auxin-responsive GH3 family protein
		AT2G23170	Auxin-responsive GH3 family protein
		AT4G27260	Auxin-responsive GH3 family protein
		AT5G13320	Auxin-responsive GH3 family protein
	**SAUR**	AT4G22620	SAUR-like auxin-responsive protein family
		AT5G50760	SAUR-like auxin-responsive protein family
		AT2G45210	SAUR-like auxin-responsive protein family
**SA**	**TGA**	AT1G08320	bZIP transcription factor family protein
	**PR-1**	AT4G33720	CAP (Cysteine-rich secretory proteins, Antigen 5, and Pathogenesis-related 1 protein)
		AT2G14610	Pathogenesis-related gene 1
**Cytokinine**	**AHP**	AT3G21510	Histidine-containing phosphotransmitter 1
**ABA**	**PP2C**	AT5G59220	Highly ABA-induced PP2C gene 1
**ETH**	**ERF1/2**	AT3G23240	Ethylene response factor 1
		AT5G47220	Ethylene responsive element binding factor 2
**BR**	**TCH4**	AT5G57560	Xyloglucan endotransglucosylase/hydrolase family protein
**JA**	**JAZ**	AT1G17380	Jasmonate-zim-domain protein 5
		AT1G19180	Jasmonate-zim-domain protein 1
		AT5G13220	Jasmonate-zim-domain protein 10

Table S3. Primer sequences used in the present study.

3	Gene name	*Primer*	Amplicon Length (bp)	Amplification efficiency (%)	Primer *Tm* (°C)
**RT-qPCR**	*ChACT-qFor*	*GTCCTACGAGCTTCCCG*	201	90.27	51.9
	ChACT-qRev	*ATGGTGGTACCACCAGAC*			50.3
**RT-qPCR**	*TUB2-qFor*	*ATCCGTGAAGAGTACCCAGAT*	144	104.87	52.4
	TUB2-qRev	*AAGAACCATGCACTCATCAGC*			52.4
**RT-qPCR**	*CH63R_01708 -qFor*	*GTCAAAGTCACTACGGCTGC*	115	95.33	53.8
	CH63R_01708-qRev	*GCTCCTCGACGATTCCCATA*			53.8
**RT-qPCR**	*CH63R_03174-qFor*	*CTACCCTGCCAGCATCTTTG*	268	93.72	53.8
	CH63R_03174-qRev	*GATGACCTTGACCTTGGACG*			53.8
**RT-qPCR**	*CH63R_01918-qFor*	*CAGCAGAATCTCCCAGGCTA*	193	99.48	53.8
	CH63R_01918-qRev	*GACAAGGTCTTCGGCAACAC*			53.8
**RT-qPCR**	*CH63R_09049-qFor*	*GTCCTCCAAGTCCTCTTCCC*	194	94.84	55.9
	CH63R_09049-qRev	*AGTAGTCGCTGTTGAGGATGT*			52.4
**RT-qPCR**	*CH63R_03670-qFor*	*TCACCGTTACCGTATCCACC*	132	98.9	53.8
	CH63R_03670-qRev	*TCTTGTAGCAGTCCAGACCG*			53.8
**RT-qPCR**	*CH63R_06437-qFor*	*AGAGACCGACACGCTGATTA*	135	101.23	51.8
	CH63R_06437-qRev	*CGGGCTTCCAACATTGTGAT*			51.8
**RT-qPCR**	*AT3G60120.1-qFor*	*AGGTGTAAGAGGTGGTTCGG*	217	107.88	53.8
	AT3G60120.1-qRev	*GGCTTGTTGGATGCTTTGGA*			51.8
**RT-qPCR**	*AT5G50760.1-qFor*	*GCTTTGGCGGATATGAAGTCT*	90	98.62	52.4
	AT5G50760.1-qRev	*GCAAATCGGGGAATTCGTGA*			51.8
**RT-qPCR**	*AT5G59220.1-qFor*	*CGAGCTAGTCCGTGAAGAGT*	93	96.63	53.8
	AT5G59220.1-qRev	*AACGCAACAACCTCCATGTC*			51.8
**RT-qPCR**	*AT1G64780.1-qFor*	*CGCAGGTTCAGGAGTTGTTC*	146	102.39	53.8
	AT1G64780.1-qRev	*TACCAAGCACGACAAGGGAT*			51.8
**RT-qPCR**	*AT5G58310.1-qFor*	*GCTGGCTAATTCACTGAGGG*	153	91.9	53.8
	AT5G58310.1-qRev	*TGATCGTTGGGAAGTTGGGA*			53.8
**RT-qPCR**	*AT4G35090.1-qFor*	*ACAACTCCTCCATGACCGTT*	92	98.82	51.8
	AT4G35090.1-qRev	*CCGTTCCCTGTCGAAATTGG*			53.8
**PCR**	*THR1upF*	*ACGACGGCCAGTGCCAA-GCTTCCGCAAAACACGAGGATTTC*	1542		60.5
	THR1upR	*GACCTGCAGGCATGCAA-GCTTCTTGAAA-GATTGTGGGGGGG*			61.5
**PCR**	*THR1dsF*	*CCGGGTACCGAGCTCGAATTCAC-CACATTCAACCACCTAACTGTAGT*	1542		60.6
	THR1dsR	*TATGGAGAAACTCGA-GAATTCGCGACAAGCAGAAAC-GTGTGG*			63.9
**PCR**	*THR1probeF*	*CGCGTCGGCGGATG*	700		58.0
	THR1probeR	*CCCGGATGCCCGTG*			56.2
